# Fostering Positive Communities: A Scoping Review of Community-Level Positive Psychology Interventions

**DOI:** 10.3389/fpsyg.2021.720793

**Published:** 2021-09-20

**Authors:** Corentin Montiel, Stephanie Radziszewski, Isaac Prilleltensky, Janie Houle

**Affiliations:** ^1^Department of Psychology, Université du Québec à Montréal, Montréal, QC, Canada; ^2^School of Education and Human Development, University of Miami, Coral Gables, FL, United States

**Keywords:** positive psychology, community psychology, well-being, communities, intervention, scoping review

## Abstract

Historically, positive psychology research and practice have focused on studying and promoting well-being among individuals. While positive psychology interventions focusing on the well-being of communities and marginalized groups have recently been developed, studies reporting on their nature and characteristics are lacking. The aim of this paper is to examine the nature of community-level positive psychology interventions. It reviews the target populations, intervention modalities, objectives, and desired effects of 25 community-level positive psychology interventions found in 31 studies. This scoping review shows that community-level programs based on positive psychology vary greatly in all these aspects. However, most interventions are aimed at individual-level changes to achieve target group outcomes. Contextual issues such as social conditions, values, and fairness affecting well-being are rarely considered. Discrepancies between community-level positive psychology interventions and community psychology in terms of values and social change are discussed.

## Introduction

### Positive Psychology

In recent decades, positive psychology has been one of the fastest growing disciplines with regards to well-being research and practice (Ivtzan et al., 2016). Positive psychology evolved in reaction to growing frustrations with the limitations of traditional models of psychology. In contrast to prevalent paradigms, positive psychology focuses on optimal human flourishing (Seligman and Csikszentmihalyi, 2000). Although numerous descriptions of the field can be found, core themes and consistencies have been identified by Linley et al. (2006). In their view, positive psychology is the scientific study of optimal human functioning. The study of positive psychology operates at three distinct levels: (1) the subjective level, (2) the group, or community level, and (3) the individual level (Kim et al., 2012). Although the subjective level is focused on positive emotions such as well-being, life satisfaction, happiness, optimism and flow, the group level emphasizes civic virtues, social responsibilities, nurturance, altruism, civility, tolerance, work ethics, positive institutions, and other factors that contribute to the development of citizenship and communities (Boniwell, 2006). Finally, the individual level is about ways to become a better person, focusing on human virtues, and character strengths. These virtues are perceived to be core human characteristics valued in most cultures around the world. Character strengths are psychological processes or mechanisms through which a particular virtue is given expression (Peterson and Seligman, 2004, p. 13). Twenty-four character strengths compose six virtues: wisdom and knowledge, courage, humanity, justice, temperance, and transcendence.

### Positive Psychology Interventions

Initially, positive psychology interventions were defined as “[. . .] treatment methods or intentional activities that aim to cultivate positive feelings, behaviors, or cognitions” (Sin and Lyubomirsky, 2009; p. 468), but not “[. . .] programs, interventions, or treatments aimed at fixing, remedying, or healing something that is pathological or deficient” (Sin and Lyubomirsky, 2009; p. 468). While this initial definition focused on increasing positive elements, researchers have since allowed for a broader view of positive psychology interventions and included effects on negative aspects such as weaknesses, difficulties, and unhappiness (Schueller and Parks, 2014; Worth, 2020). Researchers have investigated the possible effects of positive psychology interventions in a wide array of outcomes. Meta-analyses have shown that positive psychology interventions may be effective in fostering character strengths such as gratitude, kindness, humor, and hope (Carr, 2011), and enhancing well-being outcomes (Sin and Lyubomirsky, 2009). They have also reported positive impact of such interventions on the reduction of depressive symptomatology (Bolier et al., 2013). Finally, outcomes such as work-life fit, leadership skills, and work performance have also been studied in school and work settings (Waters, 2011; Meyers et al., 2013).

Although the field has historically mostly focused on individual well-being (Schueller, 2009; Di Martino et al., 2018b), communal and national well-being has also been considered. Seligman has indeed argued that positive psychology aims to create “a psychology of positive human functioning that achieves a scientific understanding and effective interventions to build thriving individuals, families, and communities” (Seligman, 2002, p. 7). There is, however, very little information on how to achieve this higher-level well-being. Interestingly, Seligman and Csikszentmihalyi (2000) have given some insight on what group-level positive psychology should aim for:

At the group level, it is about the civic virtues and the institutions that move individuals toward better citizenship: responsibility, nurturance, altruism, civility, moderation, tolerance, and work ethic. (p. 5)

Although positive psychology interventions have largely targeted individual-level traits, such civic virtues have been neglected. Research and practice in fostering citizenship is lacking in positive psychology. Critics have been arguing for years that, while setting out to counterbalance traditional psychology, positive psychology ended up mirroring many of its facets, such as the focus on individual-level factors (Worth and Smith, 2018). Moreover, though programs have been deployed in a wide range of clinical, school, and work settings, community-based interventions are rare. It is evident that the field of positive psychology has focused almost exclusively on individual-level well-being and ignored community, nation and group levels of research, and intervention. There is, however, an interest in the application of positive psychology concepts in groups, as demonstrated by the growing number of studies in workplace and educational settings.

### Communities

To study community-level interventions, it is necessary to define what a community is. This is not a trivial task. The term *community* is widely used but has never received an accepted definition (Cohen, 1985; Trickett and Espino, 2004). Chavis and Newbrough (1986) proposed that a sense of community is the organizing concept for the psychological study of community. Warren (1978) has highlighted six different notions: the community as space, as people, as shared values and institutions, as interaction, as a distribution of power and as a social system. McMillan and Chavis's (1986) model describes communities as comprising only four perceptual components: (1) membership (belonging to a community); (2) influence (mattering to the community); (3) integration and fulfillment of needs (the community meeting one's needs); and (4) shared emotional connection (having shared interests/experiences with other community members). These characteristics tend to be associated with strong communities, healthy, and happy individuals (e.g., Davidson and Cotter, 1991; Fisher et al., 2002; Hystad and Carpiano, 2009; Molix and Nichols, 2013).

McLeroy et al. (2003) propose that, regarding community-based interventions, communities can either be defined in terms of setting, target of change, resources, or agent. These are in line with the definition of Vaandrager and Kennedy (2017), in which a community can be understood as a place, an individual and collective identity, a social entity, and a collective action. A participatory public health study, aimed at defining the concept of community, concluded that it was “a group of people with diverse characteristics who are linked by social ties, share common perspectives, and engage in joint action in geographical locations or settings” (MacQueen et al., 2001, p. 1929). Ultimately, researchers now seem to privilege a definition of community in terms of geographical area or in terms of relational group with common interests or collective identity (e.g., Netting et al., 2013). Based on the existing literature, the definition of community used in this paper is *groups of people who share distinctive characteristics associated with common interests or identities*. These could be solely geographical, such as residents of the same neighborhood, or sharing joint action, like marginalized and at-risk groups.

### Critical Positive Psychology

Some researchers have described an elitist approach to positive psychology, which focuses on WEIRD (Western, Educated, Industrialized, Rich, and Democratic) groups, with little recognition of the influence of social context, and social determinants of health and well-being (e.g., Banicki, 2014; Brown et al., 2018; Hendriks et al., 2019). Although critical psychologists concur with positive psychologists in that people are resilient and have inner strengths to pursue purpose and meaning in life, the former critique the latter for their lack of attention to power differentials and social injustice. Critical psychologists argue that positive and mainstream psychologists neglect the sociopolitical context of people's lives, assuming, wrongly, that anyone with the right skills can overcome any sort of adversity. That is simply incorrect (Brown et al., 2018). Many people succumb to social adversity, and only very few are able to remain psychologically unscathed from the injuries of injustice, oppression, and discrimination (Prilleltensky, 1994, 2008, 2012; Prilleltensky and Nelson, 2002).

### Integrating Positive and Community Psychology

There are similarities but also meaningful differences between positive and community psychology. Both fields share a strength-based approach and reject the definition of mental health as the absence of illness (Schueller, 2009). Practitioners of both fields believe that human beings are capable of self-determination and autonomy. They also share the assumption that it is better to build on assets rather than deficits. But, the similarities pretty much end there. Community psychologists are very concerned with the impact of sociopolitical conditions on personal, relational, organizational, and community well-being; whereas positive psychologists remain largely silent on these issues (Brown et al., 2018; Di Martino et al., 2018b). In addition, community psychologists are concerned with challenging conditions of injustice, whereas positive psychologists are somewhat indifferent to the societal status quo (Di Martino et al., 2018b; Prilleltensky and Prilleltensky, 2021). Researchers using a community psychology lens seek to integrate context, social justice, and values in their work (Di Martino et al., 2018b). As such, they promote the involvement of disadvantaged communities in creating solutions to their own problems, building supportive structures to help people in need (Nelson et al., 2014). Positive psychologists, in turn, shy away from creating alternative social settings or engaging grassroots organizations. By and large positive psychology remains the province of WEIRD people.

These differences notwithstanding, it is important to understand how positive psychology can contribute to community well-being. This is difficult to ascertain without a review of the field. Perhaps there are positive psychology interventions that can be incorporated into community programs. We cannot provide a clear answer to that question without a thorough examination of the existing evidence. In light of this rationale, the goal of this paper is to critically examine and present a review of the current literature on positive psychology interventions in the context of communities. By mapping out the literature (Munn et al., 2018), we seek to identify the gaps between positive and community psychology. The process followed the methodological framework for scoping reviews proposed by Arksey and O'Malley (2005) and further advanced by Levac et al. (2010).

### Objective

The current study aims to provide a description of the potential of positive psychology interventions in communities by presenting the characteristics of interventions implemented within this context. Following Munn et al. (2018) recommendations on evidence synthesis approaches, a scoping review was deemed appropriate to meet these exploratory objectives.

## Methods

### Selection Criteria

As positive psychology interventional outcomes are numerous and used in many fields of practice and research, we solely considered papers explicitly mentioning their intervention being based on positive psychology theory or concepts. Doing so ensured a common theoretical background shared by the interventions reviewed and allowed researchers to be free from making choices to determine what is or is not positive psychology, a challenging process reported in other reviews (e.g., Meyers et al., 2013). Based on the work of Hillier-Brown et al. (2014), community-level interventions were defined as group-based well-being promotion, prevention, education, advice, policy or subsidy interventions, or interventions conducted in a community setting (e.g., churches, community centers, neighborhoods).

Studies were therefore included if they: (1) addressed a community-level intervention; (2); linked the program theory to positive psychology concepts and theory; (3) used measures of individual, group or community-level well-being; (4) were in English. Studies were excluded if the program was: (1) a psychotherapy/counseling intervention; or (2) delivered through an educational or a workplace setting. The rationale behind the exclusion of educational or workplace institutions is that positive psychology interventions in these unique contexts have specific target populations of students or workers, have very distinct objectives linked to their setting, and have been the subject of ample scientific research and publication in the field (see Waters, 2011; Meyers et al., 2013). Theoretical papers with no empirical investigation were also excluded.

### Search Strategy

**Figure 1:**
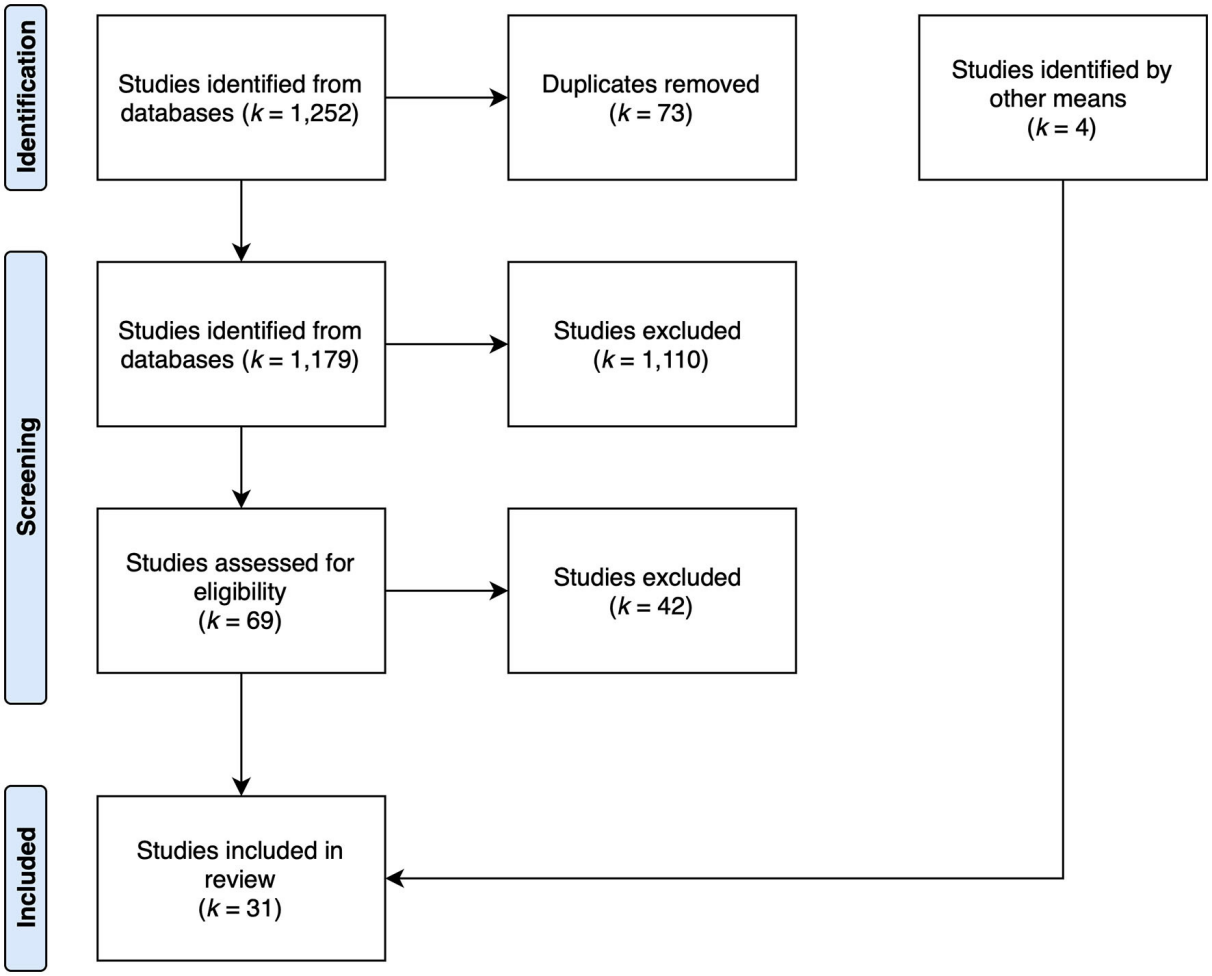
Flow chart depicting the process of selection of paper for final analysis.

The databases PubMed and PsycINFO were searched for publications until January 2021. The initial search resulted in 1,252 hits. A number of 73 duplicates were removed for a total of 1,179 publications to review (see Figure 1). The search strategy was intentionally broad in order to identify potential interventions which did not mention positive psychology in the abstract but linked its theoretical background in the article (see Table 1).

**Table 1:** Keywords constituting the syntax entered in the databases.

**Included**	**Included**	**Included**	**Excluded**
“Positive psychology” OR “Positive mental health” OR “Positive environment” OR “Strength-based” OR “Asset-based”	“Intervention” OR “Program” OR “Action research” OR “Appreciative inquiry”	“Community” OR *Related MeshTerms/IndexTerms*	“School” OR “College” OR “University” OR “Workplace”

Abstracts were first scanned by two graduate students (CM and SR); those satisfying the criteria were set aside for full-text reading. The two authors first reviewed the same randomly selected 100 abstracts (around 8%) with a criteria grid they established. They then compared their results. A preliminary inter-rater agreement of 85% was achieved. Differences were discussed and agreements were achieved. The grid used was refined to specify ambiguous elements (such as a shared definition of the concept of community) before each author went to read half of the remaining abstracts. Psychotherapy and counseling interventions were excluded at this stage. Interventions based on positive aging (Hill, 2005; Hill and Mansour, 2008), an extension of the positive psychology movement focusing on issues specific to old age (Hill, 2011), were included. However, positive youth development interventions were not included since, although conceptually similar to positive psychology, the field is not considered part of positive psychology (see Lerner, 2005). All full articles were read by both CM and SR. Most of the studies that were excluded from our analysis were theoretical in nature and did not involve the empirical investigation of well-being interventions. A total of 27 studies fit the criteria applied. The authors identified 4 additional relevant publications cited in the articles read for a final count of 31 articles included in the review.

### Analysis and Synthesis

For each study, interventional target populations, modalities, intervention objectives, desired effects, and reported effectiveness were reviewed. Both reviewers independently extracted data from half of the studies. Theoretical background and participatory methods were also assessed following discussions between them. Interventional outcomes measured were separated in primary and secondary outcomes, when specified. Outcomes reported through the use of a qualitative research method were identified as “emerging” with quotation marks. The numerous desired effects reviewed were grouped into types of well-being following the classification proposed in the I COPPE scale (Prilleltensky et al., 2015). The I COPPE types of well-being were chosen as they include both individual and community well-being, the latter often being omitted or underdeveloped in other well-being conceptualisations. The I COPPE scale consists of overall well-being and six domains of well-being: interpersonal, community, occupational, physical, psychological, and economic (see Table 2). While the I COPPE scale itself focuses on classification of subjective well-being, the authors of this article deemed appropriate to use its terminology to group desired effects into meaningful categories of well-being being targeted. Although the inclusion of a distinct spiritual well-being category has been proposed in the past (Di Martino et al., 2018a), it can also be considered as part of psychological well-being (Bozek et al., 2020). Due to the lack of consensus and the potential difficulty in separating spiritual well-being to the spirituality character strength, this type of well-being was not included in the current classification.

**Table 2:** Classification of well-being outcomes derived from the I COPPE scale.

**Well-being**	**Classification**
Overall	Outcomes related to non-specified, general or overall well-being
Physical	Outcomes related to physical health and wellness
Psychological	Outcomes related to emotional life
Interpersonal	Outcomes related to relationships
Occupational	Outcomes related to occupations
Economic	Outcomes related to financial situation
Community	Outcomes related to the community

Well-being was categorized according to the definitions reported in the studies reviewed. When there was no indication of the type of well-being measured, outcomes and instruments were used to categorize well-being variables according to our classification. Finally, character strengths were identified following Peterson and Seligman's definitions (Peterson and Seligman, 2004). This included outcomes not explicitly named as character strengths but comprised in the definitions given by the authors.

## Results

In order to document community-level positive psychology interventions' characteristics, we reviewed the year of publication and the country of origin, the program target populations, intervention objectives, outcomes, effectiveness and modalities, theoretical background and participatory methods. With the exception of the year of publication and country of origin of the included studies (*k* = 31), the results are grouped and presented by program/intervention (*n* = 25).

### Description of Included Studies

Table 3 provides a description of included studies (*k* = 31) in terms of publication year and country of origin. The majority of included studies were published between 2015 and 2019 (67.7%) whereas another 19.4% were published in the last 2 years. Around 29.0% of studies included were conducted in Hong Kong (China). However, the majority of these studies were conducted by the same group of authors and pertained to the same program series (i.e., FAMILY programs). While another 32.2% of the studies were conducted in the United States, some articles also came from Australia and Italy. A minority of studies were conducted in countries such as Brazil, Canada and Ghana.

**Table 3:** Publication year and country of origin of included studies.

**Included articles (*k* = 31)**		***k***	**%**
Publication year	2010–2014	4	12.9
	2015–2019	21	67.7
	2020-present	6	19.4
Country	United States	10	32.2
	Hong Kong, China	9	29.0
	Australia	2	6.5
	Italy	2	6.5
	Brazil	1	3.2
	Canada	1	3.2
	Ghana	1	3.2
	India	1	3.2
	Iran	1	3.2
	Spain	1	3.2
	Taiwan	1	3.2
	United Kingdom	1	3.2

### Target Population

As seen in Table 4, programs included in the analyses targeted various populations. A good proportion of programs reviewed targeted older adults (40.0%), which were often residents of nursing homes. Five (20.0%) programs targeted people with different physical conditions, including multiple sclerosis, metabolic syndrome, Parkinson's disease and having received a recent transplant. Some interventions targeted groups with low socioeconomic resources (16.0%), such as individuals living in poor rural communities, homeless female youth, and residents of a low socioeconomic neighborhood. Three related programs (i.e., the Family Kitchen series) targeted families (12.0%). Others targeted what was described as at-risk groups (8.0%), such as female victims of intimate partner violence and mental health service users. Churchgoers and unpaid carers of dependent people made up the target populations of the two remaining programs.

**Table 4:** Target population of the programs reviewed (*n* = 25).

**Target population**	**n**	**%**
Elderly people	10^*^	40.0
People with a physical health condition	5	20.0
Groups with low SES	4	16.0
Families	3	12.0
At-risk groups	2	8.0
Unpaid carers of dependent people	1^*^	4.0
Churchgoers	1	4.0

* *One of the programs targeted two different groups (Bartholomaeus et al., 2019)*.

### Intervention Objectives

Supplementary Table 1 provides a summary of the intervention objectives found in studies reviewed. Studies reviewed had numerous intervention objectives, which were mostly geared toward increasing well-being, promoting functioning, or reducing symptomatology. Increasing or promoting well-being was part of the target objectives of 13 programs (52.0%). This included family, mental, social, psychological, positive, and subjective types of well-being. Objectives mentioning health (e.g., increasing health behaviors, promoting mental health, positive mental health, improving health promotion) were part of six (24.0%) interventions. Other, more precise, health-related outcomes such as improving markers of hypothalamic-pituitary-adrenal axis and inflammation were also present. Five (20.0%) programs directly aimed at fostering character strengths and related assets such as resilience and optimism, gratitude, grace, self-forgiveness, and hope. Reducing depressive symptomatology was the target objective of two other programs (8.0%). Other program objectives included happiness (8.0%), quality of life (8.0%), family communication (8.0%) or family relationships (4.0%), physical activity (4.0%), perceived social isolation (4.0%), self-efficacy and morale (4.0%), working memory (4.0%) and psychological capital (4.0%).

Some of the intervention objectives reviewed aimed at countering or alleviating the loss of well-being associated with the condition of certain groups. For example, multiple studies targeting older adults mentioned the reduced happiness and well-being associated with aging (e.g., Ho et al., 2014; Bartholomaeus et al., 2019). Interventions with low-income populations mostly aimed at promoting well-being outcomes and building strengths to prevent mental health symptomatology associated with economic and living conditions (e.g., Hou et al., 2016; Rew et al., 2016; Sundar et al., 2016). In the context of populations with a physical health condition, positive psychology interventions were mostly used to improve recovery outcomes, and reduce associated psychological distress through increased positive psychology states (e.g., Millstein et al., 2020; Amonoo et al., 2021). In the case of chronic illness, objectives could also be associated with management and coping, rather than recovery (e.g., Nikrahan et al., 2016; Murdoch et al., 2020). The program series focusing on families presented clear links between their target population and their objectives of increasing family communication and well-being (e.g., Ho et al., 2016a,b,c). Finally, the rationale of the grace intervention for a group of churchgoers (Bufford et al., 2017) could not be determined from the information provided in the article.

### Intervention Outcomes

Desired effects of interventions were assessed in order to better comprehend how interventions were to achieve their objective. Our review suggests that community-level positive psychology interventions targeted many different outcomes. A total of 200 intervention outcomes were identified (Supplementary Table 1). They were classified according to the type of well-being targeted and 231 types of well-being outcomes were identified, with some outcomes targeting multiple types of well-being. Table 5 provides a summary of programs with at least one target outcome of each well-being category.

**Table 5:** Number of programs with at least one target outcome belonging to the different well-being categories (*n* = 25).

**Outcomes**	n^*^	%^*^
Psychological well-being	21	84.0
Overall well-being	19	76.0
Interpersonal well-being	13	52.0
Physical well-being	12	48.0
Community well-being	2	8.0
Occupational well-being	2	8.0
Economic well-being	0	0
*Character strengths*	14	56.0

* *n = number of programs with at least one outcome belonging to the category*.

* *% = percentage of programs with at least one outcome belonging to the category*.

Psychological well-being was the most widely targeted type of well-being among the different programs. Twenty-one (84.0%) interventions had at least one targeted outcome related to this type of well-being. Anxiety and depression were some of the most frequent target outcomes, along with positive and negative affect, mental health, and resilience. General psychological well-being was also common, though its definition was varied among authors. Nineteen (76.0%) of the programs reviewed targeted at least one overall well-being variable. Life satisfaction, happiness, well-being and quality of life were among the most frequently targeted outcomes. Around half (52.0%) of the interventions targeted at least one character strength. Hope, optimism, gratitude, and spirituality were the character strengths the most often aimed at. Most programs targeting character strengths also targeted well-being outcomes. Some authors conceptualized character strengths as proximal effects of the intervention with distal well-being outcomes resulting from these changes. Others did not do such distinction and considered both types of outcomes at the same level. Twelve (48.0%) programs targeted at least one physical well-being outcome. Thirteen (52.0%) programs also included at least one interpersonal well-being target outcome. Most of these outcomes were related to family relationships, such as family harmony, family communication time, and marital satisfaction, but others were more general (e.g., social connectedness, perceived social isolation, social support). Physical well-being outcomes were varied, but the most common were general physical health, physical quality of life, sleep quality, and self-efficacy in managing a disease. Other outcomes were more precise (e.g., weight, blood pressure, HPA-axis activity markers, substance use). The remaining types of well-being outcomes accounted for a negligible proportion of targeted outcomes, with community well-being and occupational well-being targeted by 8.0% of programs each. Community well-being outcomes included environmental barriers and neighborhood walking resources, and the implementation of a community service project. Occupational well-being outcomes included occupational attainment and the theme of the “engaged life” from a qualitative study. In this case, it was reported that participants (retirees) exhibited elements of confidence, mastery, accomplishment, and involvement in activities following their participation. Finally, no program targeted an economic well-being outcome. Sixteen (8.0%) of all measured outcomes could not fit into these categories, with ten (40.0%) of the programs reviewed targeting an outcome that was not related to character strengths or types of well-being used. Most of these outcomes were related to cognition (e.g., working memory, positive thoughts), behaviors (e.g., coping strategies), or attitudes (e.g., attitudes toward psychology). Spiritual well-being made up 12.5% of non-categorized outcomes.

The evaluation of the effectiveness of interventions is presented in Supplementary Table 2. The wide variety of research designs, methodologies and statistical analyses used by the different authors has not allowed us to rigorously assess and report on the effectiveness of the different programs. Nevertheless, we have identified trends suggesting significant increases of resilience, happiness and life satisfaction, and significant reductions of anxiety/depression symptomatology following the community-level positive psychology interventions. Effects on character strengths were mixed, whereas effects on physical well-being outcomes were mostly non-significant.

### Intervention Modalities

Table 6 presents the intervention modalities of the reviewed programs. In order to attain the desired effects, most studies offered in-person activities (92.0%) whereas two programs were delivered by phone (8.0%) and one of these also gave access to a web-based participant forum (4.0%). The types of activities were similar throughout the programs. Most programs (84.0%) referred to psychoeducational components, such as lectures to define concepts, or didactic books and handouts for educational purposes. A large portion of the programs (80.0%) also focused on skill/strength training, such as breathing exercises, use of personal strengths and coping strategies, or goal setting. Most programs (80.0%) sought to capitalize on their group format by using discussions to report on one's progress since last session, explore one's understanding of themes, or to offer mutual support. Many programs (68.0%) required self-directed exercises or homework to be completed between sessions, with examples ranging from keeping a diary to record positive emotions or events, monitoring physical activity, or completing acts of kindness. A small portion of the programs (16.0%) included art-based activities, such as storytelling, collaborative song writing, or writing of a fairy tale based on one's life. Finally, a portion of programs (24.0%) offered other types of activities, which ranged from group walks to a sermon series, or optional post-training mentoring. Intensity varied considerably across programs, ranging from one 120 min core session with optional booster to a 6-months interactive program of 5 days per week.

**Table 6:** Mode of participation and type of activities of positive psychology programs reviewed (*n* = 25).

		***n***	**%**
Mode of participation	In-person	23	92.0
	Phone	2^*^	8.0
	Web	1^*^	4.0
Type of activities	Psychoeducation	21	84.0
	Skill/strength training	20	80.0
	Group discussions	20	80.0
	Self-directed/Homework	17	68.0
	Art-based	4	16.0
	Other	6	24.0

* *One program offered both phone- and web-based activities (Alschuler et al., 2018)*.

### Theoretical Background and Participatory Methods

While the vast majority of the programs were grounded in positive psychology (92.0%), two (8.0%) were based on positive aging. Interestingly, a little more than half of them (60.0%) integrated other theoretical approaches into their interventions. A portion of these multi-theoretical programs (24.0%) included cognitive behavioral therapy techniques. The others (36.0%) incorporated aspects of mindfulness, religious doctrine, holistic health, ecological model, positive youth development, stress management theory, and others.

Most programs reviewed (76.0%) did not include participatory methods. Four of the six programs that included participatory methods were implemented in the context of the FAMILY project and followed a similar structure (Ho et al., 2014, 2016a, 2020a; Zhou et al., 2016; Chu et al., 2018). In these programs, researchers gathered non-governmental organizations, schools, or social service organizations with whom families were already in contact. The research team offered “train-the-trainers” workshops so that representatives from the organizations could develop and implement brief community-based interventions focused on the targeted concepts. This allowed for the representatives to tailor the intervention to their communities' preferences and needs, while following a general implementation protocol. In the grace intervention (Bufford et al., 2017) the pastors from the two churches collaborated in designing the intervention to ensure that it corresponded to their members' beliefs and practices. Finally, the Hero Lab project (Sundar et al., 2016) is an extensive, 6-month program where initial lessons on positive psychology concepts led youth participants to develop and implement their own project in their neighborhood. The curriculum was also taught by a trained community leader of the same background in regard to faith (Hindu), language, and geography (same community).

## Discussion

The aim of this study was to review the nature of positive psychology interventions taking place in communities. The first finding of this review is that positive psychology interventions implemented in the context of communities mostly aim at increasing well-being, promoting functioning, or reducing symptomatology. These are consistent with a meta-analysis by Sin and Lyubomirsky (2009), in which the authors reviewed the effects of 51 positive psychology interventions and found support for the hypothesized favorable effect on well-being and for a mitigating effect on depression. There seems to be a consensus that positive psychology interventions do not only increase well-being through multiple theoretical pathways relying on increasing different sets of character strengths, but also improve functioning and decrease negative symptoms (see Worth, 2020), sometimes related to illness or aging. Our analysis of objectives and target outcomes showed that authors were mostly interested in distal positive effects of interventions on different types of well-being [reducing depression] rather than proximal effects on character strengths [developing optimism to then reduce depression]. Even though some positive psychology models have been proposed to explain how programs achieve their objectives (e.g., Lyubomirsky and Layous, 2013; Raymond et al., 2019), current practices make it difficult to develop a comprehensive logic model of positive psychology interventions. It has been argued, for example, that current positive psychology interventions are conceived as cohesive units of activity, which limit their development and evaluation (Raymond et al., 2019; Pawelski, 2020). Through our scoping review, we found that researchers present logical connections between intervention objectives and target populations, but that there is a lack of cohesion and reasoning behind activities implemented and some of the target outcomes measured. The analysis of the different constitutive elements and processes involved in an intervention would allow for a better understanding of the specific elements essential for effective positive change in different contexts (Raymond et al., 2019; Pawelski, 2020).

Interestingly, most outcomes were considered either overall, physical, interpersonal or psychological well-being, or a character strength. This is somewhat coherent with reviews of positive psychology interventions in organizational settings, in which overall and occupational well-being were targeted (see Meyers et al., 2013), and school settings, in which character strengths and psychological well-being were the most targeted (see Waters, 2011). What is evident from this review is that, while the interventions reviewed did take place in community settings, only one (Hero Lab) was designed to improve the actual community. The vast majority of interventions took place in community and group settings, but the target of the interventions were individuals. This stems from the fact that the interventions developed are modeled on individual positive psychology interventions consisting of weekly sessions focused on psychoeducation (see Parks and Titova, 2016), effectively resulting in a group version of these programs. Communities are treated as passive samples of homogeneous groups of participants with shared characteristics rather than active actors who can participate to better their situation. This is clear in many of the intervention objectives aiming at promoting well-being outcomes and building strengths to prevent mental health symptomatology associated with economic and living conditions rather than working on changing these conditions. There is a meaningful difference between interventions taking place in the community, and programs aimed at improving the community. This review provides clear evidence that most positive psychology interventions address the former and neglect the latter.

It is possible that positive psychologists surmise that communities and organizations will become better if the individuals residing in them become happier and healthier. But this is a problematic assumption. Individual happiness does not necessarily translate into happier organizations and communities. It is true that happier individuals are more tolerant and express more gratitude, creating a gentler psychosocial environment, but this is not the same as creating settings based on fairness and equity. There is abundant evidence that many social structures perpetuate discrimination against people with disabilities, ethnic minorities, and LGBTQ individuals (Prilleltensky and Nelson, 2002; Denison et al., 2020; Prilleltensky and Prilleltensky, 2021). Some of the barriers to the well-being of these individuals are not interpersonal, but structural. None of the interventions reviewed address power differentials, social injustice, or oppression. In that regard, the critique leveled against positive psychology, that it is similar to mainstream psychology in its individualistic orientation, is borne out by our results (Brown et al., 2018; Di Martino et al., 2018b).

Having said that, it is possible to build on these positive psychology interventions as a first step in the route toward community well-being. It can be argued that happier and healthier individuals will be better prepared to engage in social change efforts. From this perspective, positive psychology interventions can become a first step in preparing people to collaborate with others in the struggle for social justice. Happier people are usually more productive and collaborative (Prilleltensky, 2016), a great start to coalition building. But if positive psychology interventions begin and end with the individual, and ignore the collective fate of communities, their social and global impact will be limited. Evaluating and even challenging collective norms is especially important in the context of oppressive communities, where minorities are persecuted because of religious or other prejudices (Sandler, 2007). For example, sexual minorities are often discriminated in some countries (Harper and Schneider, 2003). It may be argued that challenging oppressive social norms is beyond the scope of positive psychology, but it is difficult to promote well-being, at any level, without considering power differentials and exclusionary cultural practices (Prilleltensky, 2001, 2008).

It is also worth noting that very few of the interventions reviewed were participatory and collaborative in nature. They retained the expert model where professionals taught or guided a group of vulnerable individuals in a series of exercises. In community psychology, a participatory approach is valued because it is empowering and it builds citizenship and civic virtues. The interventions described here follow closely the medical model in which an expert imparts advice to a relatively passive recipient, a sharp contrast to community psychology values and ethos. A participatory approach is also particularly favored to recognize and build on existing strengths toward promoting social change (Israel et al., 2013). Although values are often mentioned in reviewed studies through the universal *Values In Action* model of character strengths (Peterson and Seligman, 2004), the focus is on measuring individual participants' values. This concern does not extend to researchers, as few, if any, of the articles mention the values that frame the study and the context of the intervention. From a community psychology standpoint, we would argue that values of trust, reciprocity, and equity are central in forming positive communities (Arcidiacono and Di Martino, 2016; Di Martino et al., 2018b).

Many examples of ways to foster positive nations and communities through supportive structures and institutions are discussed in Marujo and Neto (2014) book Positive Nations and Communities. In that book, the focus is on how character strengths and other positive psychology concepts could constructively contribute to building a more just and positive society. Historical and sociopolitical events such as South Africa's Truth and Reconciliation process, the collapse of Portugal's first Republic, Namibia's independence and the European Football Championship are thoroughly explained and discussed through the lens of positive psychology. Their contribution to the cultivation of positive communities is also addressed. The different authors illustrate how citizens' character strengths and well-being may be increased at a macro-level through various means such as festive events (Proyer et al., 2014), social reconciliation processes (Perstling and Rothmann, 2014; Wissing and Temane, 2014), legislation (Perstling and Rothmann, 2014), direct democratic participation and local autonomy (Lopes et al., 2014).

On a smaller scale, community interventions based on positive psychology concepts and theory would involve a good proportion of community members and aim to improve social capital among them through collective projects where they can express their gratitude and build on their character strengths and assets. They would target the improvement of the living conditions of these community members (i.e., the social determinants of health) by increasing access to green spaces, places to meet and play, and jobs where they can thrive, for example. Elements such as context, social justice and values should be taken into consideration. Such interventions therefore take time. Indeed, it is impossible to improve community well-being with a few group workshops over a short period of time.

As positive and community psychology share a common goal (i.e., to improve human well-being by gaining understanding of the psychological processes that promote well-being) and if our goal as psychologists is to work toward producing the largest benefit for most individuals (Kelly, 1971), then both community and positive psychologists have much to gain through communication and collaboration (Schueller, 2009). Nonetheless, this scoping review reveals an important knowledge gap that could guide future studies. It is crucial to move the discipline of positive psychology to a higher level of complexity where social change is also considered rather than solely focusing on individual change. With such a perspective, positive psychology has the potential to increase the well-being of more people and contribute to just societies.

This scoping review is, to our knowledge, the first exploration of community-level positive psychology interventions. It provides a worthwhile and detailed summary of intervention background, modalities, and objectives. We believe its contribution to the field to be significant, as it allows for a better comprehension of theory and practice in the field of well-being. In doing so, it strives to move toward a closer collaboration between positive and community psychology.

## Limitations

There are a few limitations worth noting about this study. First of all, it included only interventions that were published in English and the interventions reviewed here come from a relatively small number of countries. Second, there are possibly many community change efforts that use positive psychology interventions but do not frame their work that way. For example, Asset Based Community Development has many similarities to some of the interventions described here, especially the promotion of empathy and kindness (Block, 2009; McKnight and Block, 2010). Third, well-being was categorized according to the terminology used in the studies reviewed. This created some discrepancies as some authors reported targeting general well-being but used specific subscales of well-being instruments aimed at assessing solely one domain of well-being (e.g., interpersonal). While it was sometimes clear that the instrument used did not assess the variable of interest, it was still categorized according to the author's intent. It is important to keep in mind that the authors of the present study exercised discretion when some outcomes were not adequately described. Finally, the results of the effectiveness assessment we included in this review should be interpreted with precaution, as the current review included a high number of pre-experimental studies lacking a control group. Studies with control groups frequently reported significant main effects but rarely obtained time x group interactions effects. Therefore, a meta-analysis is necessary to rigorously examine the effectiveness of community-level positive psychology interventions.

## Conclusion

This scoping review revealed that positive psychology interventions taking place in the community are rich in content and delivery method. However, they focus on the individual level and aim to improve society one person at a time. The many interventions reviewed do not address contextual factors but rather individual-level phenomena. While useful to the individual participating in the program, structural factors that enable or inhibit personal, group, or communal well-being, such as unequal distribution of resources or discrimination, are not addressed by positive psychology interventions.

## Author Contributions

This manuscript evolved from a 2019 symposium presentation involving CM, IP and JH. CM conceived the presented idea with supervision from JH. CM and SR performed the literature review and analysis, with input from IP and JH on the analysis and interpretation of the data. CM and SR wrote the manuscript with support from IP and JH. All authors approved the final draft.

## Conflict of Interest

The authors declare that the research was conducted in the absence of any commercial or financial relationships that could be construed as a potential conflict of interest.

## Publisher's Note

All claims expressed in this article are solely those of the authors and do not necessarily represent those of their affiliated organizations, or those of the publisher, the editors and the reviewers. Any product that may be evaluated in this article, or claim that may be made by its manufacturer, is not guaranteed or endorsed by the publisher.
