# Supplementary material for: Fostering Positive Communities: A Scoping Review of Community-Level Positive Psychology Interventions
Source: Front Psychol. 2021 Sep 20;12:720793. doi: 10.3389/fpsyg.2021.720793 (PMC8488140; doi:10.3389/fpsyg.2021.720793)
Supplement: Supplementary file 1 [file Table_1.pdf]

Supplementary Material

Supplementary Table 1.

Summary of studies reviewed (*k* = 31, *n* = 25).

Program	Author (year), Location	Target community/ group	Intervention objective	Type of intervention/intervention modalities	**Primary**, secondary intervention outcomes measured or "emerging"	Type(s) of intervention outcomes
Everyday Matters	Alsculer et al. (2018), United States	Older adults with multiple sclerosis	Support ongoing happiness	Six 90-min sessions on psychoeducation and brief skills training in positive psychology topics (e.g., goal setting, happiness habits, building social connections, etc.) delivered via group phone teleconference and supported with a web-based group page for participants.<br><br>The program was modelled on resilience interventions, focusing on optimism, happiness, and capitalizing on opportunities for forward movement and positive outcomes. It also included special themes such as attending more closely to planning or goal setting and identifying more specifically how to implement program content in the face of adversity.	**Psychological resilience**<br>Depression<br>Anxiety<br>Satisfaction with social roles<br>Positive affect and well-being<br>Happiness<br>Self-efficacy in managing disease	**Psychological well-being**<br>Psychological well-being<br>Psychological well-being<br>Interpersonal well-being<br>Overall well-being<br>Overall well-being<br>Physical well-being
N/A	Amonoo et al. (2021), United States	Adults who had a hematopoietic stem cell transplantation	Improve psychological well-being and health behaviors	Eight-week program consisting of individual positive psychology exercises (e.g., gratitude letter, performing acts of kindness, recalling past successes, personal strength assessment, recalling of recent positive events) followed by brief discussions during 30-min group calls led by an interventionist. Groups comprised 3–4 participants, assigned by the research team.	Happiness<br>Optimism<br>Positive and negative affect<br>Social support<br>Coping strategies<br>Depression<br>Anxiety<br>Resilience<br>Gratitude<br>Physical and mental health	Overall well-being<br>*Character strength*<br>Psychological well-being<br>Interpersonal well-being<br>N/A<br>Psychological well-being<br>Psychological well-being<br>Psychological well-being<br>*Character strength*<br>Physical well-being<br>Psychological well-being
Inspired Life Program	Appiah et al. (2020), Ghana	Adults living in poor rural communities	Increase positive mental health	Ten 120-min weekly sessions for groups of around ten people. Activities included mini-lectures, discussions, and exercises on concepts such as personal strengths, self-compassion, purpose in life, or positive relations. Sessions were designed to stimulate interactive discussions where participants took turns to share their views and ask questions. Each session consisted of three parts: a review of the previous session and a discussion of home assignments; a discussion of the theme and contents of the current session and a breakout activity; and an overview of key lessons and discussion of home assignments for the following session. Sessions were facilitated by psychology graduates in participants' native language (Twi).	**Positive mental health**<br>Positive and negative affect<br>Positive thoughts<br>Coping self-efficacy<br>Depression symptoms<br>Satisfaction with life	**Overall well-being**<br>Psychological well-being<br>N/A<br>N/A<br>Psychological well-being<br>Overall well-being

N/A	Baker & Ballantyne (2013), Australia	Older adults residing in a retirement village	Increase health and social well-being	Five 90-min collaborative song writing sessions over the course of three weeks, followed by a public performance. The sessions were implemented in the village auditorium. Keyboards, guitars and songbooks were provided with assistance from music students.	"Pleasant life"	Overall well-being
					"Well-being"	Overall well-being
					"Meaningful life"	Overall well-being<br>Interpersonal well-being
					"Engaged life"	Overall well-being<br>Occupational well-being
N/A	Bartholomaeus et al. (2019), Australia	a) Older adults<br>b) Unpaid carers of dependant people with disability or health disability	Increase well-being, resilience, optimism and reduce perceived social isolation	Eight-week face-to-face weekly well-being training sessions (90 to 120-min). Each session being designed to teach one of the ten evidence-based skills to increase well-being (e.g., growth mindset, cultivating gratitude, values-based goals). Optional post-intervention mentoring, monthly support groups and peer support were available. The training program is an adaptation of an existing resilience training program (TechWerks Resilience Training).	Well-being	Overall well-being
					Resilience	Psychological well-being
					Optimism	*Character strength*
					Perceived social isolation	Interpersonal well-being
Grace intervention	Bufford et al. (2017), United States	a) Churchgoers	Increase the experience of grace	Six weekly sermon series with the focus on grace; 2) A small-group study program with grace-focus study text; 3) A menu of personal grace practices that individual members of the congregations could carry out. Many of the grace practices could be carried out individually, while others involved relational activities with other persons. Married participants were encouraged to practice with spouse. All activities focused in some way on grace or in engaging in public or secret acts of grace.	**Grace**	***Character strength***
					Daily spiritual experiences	*Character strength*
					Religiousness	*Character strength*
					Marital satisfaction	Interpersonal well-being
					Gratitude	*Character strength*
					Positive and negative affect	Psychological well-being
					Attitudes towards psychology	N/A
					Spiritual well-being	N/A
		b) Churchgoers	Increase the experience of grace and self-forgiveness	Identical program but implemented weekly for nine weeks instead of six.	**Grace**	***Character strength***
					**Trait self-forgiveness**	***Character strength***
					Daily spiritual experiences	*Character strength*
					Religiousness	*Character strength*
					Attitudes towards psychology	N/A
					Spiritual well-being	N/A
					State self-forgiveness	*Character strength*
					View of god	*Character strength*
					Experience of god forgiveness	*Character strength*
					Responsibility for offense	*Character strength*
Lab. I Emotional-Motivational Empowerment	Cantarella et al. (2017), Italy	Older adults	Improve positive well-being, quality of life, and working memory	Six 90-min group sessions that began with breathing relaxation exercises and a follow-up on homework and then involved various activities on topics (e.g., personal growth, self-efficacy, self-acceptance). Each topic was illustrated and developed with practical assignments and the use of questionnaires. Exercises included "The good grains of the day", role-playing to identify	**Psychological well-being**	**Psychological well-being**
					Quality of life	Overall well-being
					Working memory (listening span)	N/A

				dysfunctional beliefs about yourself, and reflection on negative past experiences.		
N/A	Carson et al. (2010), United Kingdom	Community mental health team-based service-users	Increase gratitude	Two 2-hour workshops, a month of gratitude monitoring and attendance at a meal. The first workshop consisted of a presentation of the scientific literature on gratitude, and statements of what gratitude meant for the participants. The second workshop consisted of reception of feedback from participants regarding their monitoring, and discussion. They were all expected to attend both workshops and to complete a gratitude diary for a month. All participants were given a copy of a book on gratitude and happiness as well as comprehensive handouts.	**Gratitude**<br>Satisfaction with life<br>Happiness<br>Well-being<br>Psychological well-being<br>Life thankfulness	***Character strength***<br>Overall well-being<br>Overall well-being<br>Overall well-being<br>Psychological well-being<br>*Character strength*
Positive Narration Group Intervention — Older Adults	Cesetti et al. (2017), Italy	Older adults	Promote well-being	Four 2-hour weekly sessions of positive narration group intervention focused on four different emotions and four different stories and/or fairy tales. The sessions consisted of the reading of stories, group discussions aimed at recognizing, naming and analyzing the emotion involved in the fairy tale, as well as its narrative plot. Life review were proposed to recall and discuss related personal memories, emotions and experiences. Participants were then guided through their own fairy tale creation which should address the emotions previously discussed.	**Eudaimonic well-being**<br>Life satisfaction<br>Sleep quality<br>Depression<br>Social connectedness	**Psychological well-being**<br>Overall well-being<br>Physical well-being<br>Psychological well-being<br>Interpersonal well-being
Programa Vem Ser	Durgante & Dalbosco Dell'Aglio (2019), Brazil	Retirees	Improve health promotion of retirees	Six 120-min weekly sessions in groups of 5–15 participants facilitated by a trained moderator and two trained observers. Activities were organized around themes (e.g., optimism, empathy, selfcare). Activities include group debates, building the Empathy Tree, relaxation training, making CV of personal accomplishments, and the surprise balloon (learning from mistakes). Participants also had homework to do every week and techniques were based both on positive psychology and cognitive-behavioral approaches.	Empathy<br>General health (anxiety and depression symptoms)<br>Perceived stress<br>Optimism<br>Resilience<br>Life satisfaction	*Character strength*<br>Psychological well-being<br>Psychological well-being<br>*Character strength*<br>Psychological well-being<br>Overall well-being
Lighten UP!	Friedman et al. (2019), United States<br>Friedman et al. (2017), United States	Older adults	Promote psychological well-being	Eight-week program consisting of 90-min weekly group sessions delivered in community settings. The first classes focus on identifying positive experiences and introduce different conceptual domains of eudaimonic well-being. Later classes use CBT techniques to restore and sustain well-being. In-class activities are supported by at-home behavioral assignments and diaries in which participants record daily positive experiences and their reactions to them.	**Eudaimonic well-being**<br>**Hedonic well-being**<br>Depressive symptoms<br>Social connectedness<br>Physical symptoms<br>Sleep quality	**Psychological well-being**<br>**Overall well-being Psychological well-being**<br>Psychological well-being<br>Interpersonal well-being<br>Physical well-being<br>Physical well-being
Art of Happiness	Greenawalt et al. (2019), United States<br>Turner et al. (2017), United States	Older adults living in senior homes	Enhance happiness levels and overall mental well-being	8-week intervention based on the teachings of the Dalai Lama. Once per week 90-minute classes examining a different weekly topic including defining happiness, stress management, reflecting on happiness, compassion and human connection, forgiveness, transforming suffering, mindfulness, and humor. Each class session included a lecture, an in-class activity and a discussion on The Art of Happiness readings and homework	Subjective happiness<br>Perceived stress<br>Gratitude<br>Life satisfaction	Overall well-being<br>Psychological well-being<br>*Character strength*<br>Overall well-being

				assignments. Activities included Photo voice, three good things, therapy puppies, group discussion on forgiveness, savoring exercises, letter-writing and body scan meditation.	Depression	Psychological well-being
					Arousal states (mood)	Psychological well-being
					Mindfulness	Psychological well-being
Happy Family Kitchen Movement	Ho et al. (2020a), Hong Kong, China<br><br>Ho et al. (2020b), Hong Kong, China	Families	Promote well-being and health	Train the trainer workshops (2.5 hours) were offered to social service and school organizations. A manual was also provided for designing and implementing their own community program, with oversight from the research team. The organizations were randomly assigned to design interventions promoting either positive physical activity or positive healthy diet with a focus on one of three psychology themes of joy, gratitude, or savoring.<br><br>For the intervention, families participated in one 120-min. core session including group activities and homework assignments on positive psychology and physical activity. Each theme consisted of three target behaviors for participants to engage in during group activities and homework assignments so that physical activity could be encouraged, reinforced, and sustained. The different themes involved behaviors such as sharing happy experiences in physical activity with family members, appreciating the strengths of family members during physical activity and expressing one's gratitude for doing and enjoying physical activity together to family members through, paying attention to the process of physical activity. There was also a booster session (60 min.) offered one month later to consolidate knowledge and skills acquired.	Zero-Time Exercise (alone and with family member)	Physical well-being<br>Interpersonal well-being
					Balance	Physical well-being
					Muscular endurance	Physical well-being
					Family health	Physical well-being
					Family happiness	Overall well-being
					Family harmony	Interpersonal well-being
					Subjective happiness	Overall well-being
					Health-related quality of life	Physical well-being<br>Psychological well-being
					"Improved physical health"	Physical well-being
					"Enhanced family relations"	Interpersonal well-being
Happy Family Kitchen I	Ho et al. (2016a), Hong Kong, China<br><br>Ho et al. (2017), Hong Kong, China	Families	Promote family communication and well-being	Train the trainer workshops (2 days) plus a design manual were offered to social service workers so they could design and implement a community program focused on one of five positive psychology themes (gratitude, flow, happiness, health, or savoring).<br><br>Each community program consisted of two 2–3 h core sessions and a 1-h booster session, held 6 weeks after the core sessions. The two core sessions emphasized positive family communication, which were run in the form of group activities and homework assignments, while the booster session was held to consolidate the knowledge and skills from the core sessions. The group activities provided the participants with the opportunity to practice the target behaviors during the intervention program while the homework assignments reinforced the target behaviors at home. For example, the homework assignment for the gratitude intervention involved keeping a daily diary of family events that elicited gratitude, and the savoring intervention involved the practice of savoring food during family meals in order to find enjoyment in life.	Frequency of eating with family members	Interpersonal well-being
					Frequency of meal preparation with family members	Interpersonal well-being
					Family communication time	Interpersonal well-being
					Family communication quality	Interpersonal well-being
					Subjective happiness	Overall well-being
					Family health	Physical well-being
					Family happiness	Overall well-being
					Family harmony	Interpersonal well-being
					"Improved family communication"	Interpersonal well-being
					"Improved family health, happiness, and harmony"	Physical well-being<br>Overall well-being<br>Interpersonal well-being
					Gratitude behaviors	*Character strength*
					Flow behaviors	Psychological well-being

					Happiness behaviors	Psychological well-being
					Health behaviors	Physical well-being<br>Psychological well-being
					Savoring behaviors	*Character strength*
					"Improved quantity and quality of family communication"	Interpersonal well-being
					"Greater expression of appreciation towards family members"	Interpersonal well-being
					"More involvement of family members in household chores"	Interpersonal well-being
					"Theme-specific behavior changes" (flow, savoring, gratitude, happiness and health)	*Character strength*<br>Physical well-being<br>Psychological well-being
Happy Family Kitchen II	Chu et al. (2018), Hong Kong, China<br>Ho et al. (2016b), Hong Kong, China<br>Ho et al. (2016c), Hong Kong, China	Families	Promote family communication and well-being	Train the trainer workshops (2.5 hours) were offered to social service and school organizations as well as a training kit to help them plan the community program. The organizations developed the program with group activities and homework focused on one of five positive psychology themes (joy, gratitude, flow, savoring, or listening).<br>For the intervention, families participated in one 120-min. core session. There was also a booster session (60 min.) offered one month after the intervention. The goal was to promote positive family communication through family cooking and dining. Facilitators emphasized one of the following positive psychology strategies: participants were encouraged to discover the pleasures of positive family communication, express gratitude and appreciation toward family members, increase cooperation in family interactions, develop a habit of savouring both food and family interactions during family meals, or actively attend to family members' feelings, emotions and concerns.<br>The activities were developed creatively by the facilitators, such as sharing and reminiscing about a happy experience with family members and create more happy experiences by enjoying a meal together, discussing family members' contribution to family meals and other chores and expressing appreciation through words or action, learning to cooperate with each other through preparing a family meal together, guessing the ingredients of a family meal to understand the importance of savouring, or roleplaying one's response to a family members' thoughts, feelings, and concerns.	Family communication	Interpersonal well-being
					Subjective happiness	Overall well-being
					Mental and physical quality of life	Psychological well-being<br>Physical well-being
					Family health	Overall well-being
					Family happiness	Overall well-being
					Family harmony	Interpersonal well-being
					"Sense of happiness"	Overall well-being
					"Enhanced family relations"	Interpersonal well-being
					"Increased use of positive communication"	Interpersonal well-being
					"Use of age-appropriate parenting practices"	Interpersonal well-being
					"Engaging in quality family mealtime"	Interpersonal well-being
N/A	Ho et al. (2014), Hong Kong, China	Older adults	Enhance subjective well-being and	Nine-week group sessions of one and a half hours emphasized: (a) learning through experience; (b) homework assignments on positive psychology; and (c) diversification of group activities,	Depression	Psychological well-being
					Life satisfaction	Overall well-being

			reduce depression	such as the use of real-life examples, news articles, photos, and video clips. Themes included gratitude, optimism, savoring, curiosity, meaning of life, etc.	Gratitude<br>Subjective happiness	*Character strength*<br>Overall well-being
N/A	Hou et al (2016), Taiwan	Female victims of intimate partner violence	Reduce level of depressive symptoms and improve pathway component of hope	Eight weekly 90-min group sessions with a strengths-based perspective approach focusing on women's desires for their lives, aspirations, and what they are doing well. The intervention protocol was based on the aim of a support group (emotional support, education and socialisation) combined with Snyder's hope theory, which has the following three components: goals, pathways and agency. Sessions comprised the review and meaning of past intimate partner violence experiences, discovering of personal strengths, hope-related skills psychoeducation, goal setting, feedback and sharing.	Hope<br>Depression<br>Resilience	*Character strength*<br>Psychological well-being<br>Psychological well-being
Move More, Feel Well: MAPP (Motivation, Audit, and Positive Psychology)	Millstein et al. (2020), United States	Individuals with Metabolic Syndrome and low physical activity levels	Increase physical activity in people with Metabolic Syndrome	Eight-week group-based walking program with 90-min. sessions which includes a 30-min. group walk. Participants are given a manual to track their weekly activities as well as a Fitbit watch to self-monitor their physical activity. During the program, participants are encouraged to focus on their positive thoughts and feelings, especially those concurrent to their physical activity. Further, in line with motivational interviewing techniques, participants are encouraged to set "SMART" physical activity goals.	Objective physical activity<br>Self-reported physical activity<br>Environmental barriers and neighborhood walking resources<br>Barriers to completing physical activity<br>Diet (fruit and vegetable intake)<br>Fat-containing food intake<br>Dispositional optimism<br>State optimism<br>Positive affect<br>Health-related quality of life<br><br>Anxiety and depression<br>Weight<br>Blood pressure	Physical well-being<br>Physical well-being<br>Community well-being<br><br>N/A<br><br>Physical well-being<br>Physical well-being<br>*Character strength*<br>*Character strength*<br>Psychological well-being<br>Physical well-being<br>Psychological well-being<br>Psychological well-being<br>Physical well-being<br>Physical well-being
Strength, Hope, and Resource-fulness Program for people diagnosed with Parkinson's Disease (SHARP-PWP)	Murdoch et al. (2020), Canada	Individuals diagnosed with Parkinson's disease	Improve well-being and hope	Six weekly 120-min. group sessions with an emphasis on fostering hope and personal strengths while identifying personal resources. Participants take part in activities (including storytelling and art-based expressions) and discussions focused on hope and strengths in the face of Parkinson's Disease.	Health-related quality of life<br>Anxiety<br>Depression<br>Well-being<br>Hope<br>Self-efficacy for managing chronic disease<br>Relational impact<br>Emotional impact	Physical well-being<br>Psychological well-being<br>Psychological well-being<br>Overall well-being<br>*Character strength*<br>Physical well-being<br><br>Interpersonal well-being<br>Psychological well-being

					Impact on Identity/Self-Perception<br>Project a personally meaningful Future<br>Cognitive/Perceptual Shift<br>Facilitated Coping<br>Learning/Awareness	Psychological well-being<br>*Character strength*<br>*Character strength*<br>N/A<br>N/A
N/A	Nikrahan et al. (2016), Iran	Individuals living with coronary heart disease	Improve key markers of inflammation and hypothalamic-pituitary-adrenal axis functioning	Six weekly 90-min in-person group sessions. Trainer introduced strategies to boost well-being and helped to practice. Homework consisted of exercise practice. Participant were divided into three groups which each received a different positive psychology intervention.<br>1) Seligman intervention: Special emphasis on engagement and "the meaningful life". Exercises focused on enhancing positive feelings, identifying and using personal strengths and finding meaning in one's life.<br>2) Lyubomirsky intervention: Moderate overlap with the Seligman group regarding specific exercises targeting optimism and gratitude. It included exercises focusing on religion and spirituality, physical activity, and developing strategies for coping.<br>3) Fordyce intervention: Some common elements with the two previous programs in that some happiness activities focused on optimism, becoming present-oriented, and eliminating negative cognitions and feelings. In contrast to the other interventions, the Fordyce intervention focused on increasing organizational skills, setting realistic goals, and focusing on positive personality traits.	**Inflammatory marker hs-CRP**<br>Inflammatory marker IL-1<br>Inflammatory marker IL-6<br>HPA-axis activity marker CAR_g	**Physical well-being**<br>Physical well-being<br>Physical well-being<br>Physical well-being
MAPEG Program	Ramirez et al. (2014), Spain	Older adults	Increase quality of life	Nine 1.5-hour weekly training sessions based on an intervention specifically focused on forgiveness, gratitude and life review therapy. Sessions comprises themes such as an introduction to positive psychology, gratitude, autobiographical memory, life review, positive emotions in old age, the benefits of forgiving and being forgiven. The positive memories of specific events focused on childhood, adolescence and adulthood, and in the last session, this part of the program was completed by conducting a life summary. Homework assignments consisted of writing a letter of thanks, a letter of gratitude, looking for childhood photos which reminded them of happy times, remembering songs that have meaning, etc. The last session served to explain the benefits of the program, administration of the scales and questionnaires, farewells and closure of the intervention program.	State anxiety<br>Trait anxiety<br>Depression<br>General memory<br>Specific memories<br>Life satisfaction<br>Subjective happiness	Psychological well-being<br>Psychological well-being<br>Psychological well-being<br>N/A<br>N/A<br>Overall well-being<br>Overall well-being
N/A	Rew et al. (2017), United States	Homeless female youth	Enhance psychological capital and foster cognitive,	Four 1-hour sessions of a street-based intervention focused on helping the participants to look beyond their present situation to the future. Didactic information and experiential learning exercises were used with a particular emphasis on responsible	Psychological capital<br>Hope<br>Resilience	Psychological well-being<br>*Character strength*<br>Psychological well-being

			affective, and behavioral health-related outcomes	substance use and safe sexual behaviors, passive, aggressive, and assertive communication and on communicating effectively to reach their goals. The participants were also taught how to set a personal goal that could be reasonably achieved in one month's time and how to monitor their progress toward meeting that goal.<br><br>The first session included a definition of key concepts such as hope, resilience, psychological capital. The second session focused on identifying and setting attainable goals to reduce substance use and risky sexual behaviors; this session concluded with participants practicing strategies that could facilitate goal attainment. The third session included a description of communication patterns and practice in using various types of direct communication to facilitate goal attainment. The final session was a review of the previous content with a focus on setting a short-term (achievable in one month's time) goal that could enhance the participant's quality of life.	Optimism<br>Safe sex self-efficacy<br>Alcohol refusal self-efficacy<br>Social connectedness<br>Future expectations<br>Safe sex behaviors<br>Substance use<br>Goal attainment	*Character strength*<br>Physical well-being<br>Physical well-being<br>Interpersonal well-being<br>N/A<br>Physical well-being<br>Physical well-being<br>Occupational well-being
Healthy Aging Mind Body Intervention	Scult et al. (2015), United States	Older adults	Increase self-efficacy and morale	Nine 90-min weekly sessions based on a program incorporating elements from the fields of stress management, cognitive-behavioral therapy, and positive psychology. This program included sessions teaching participants methods to elicit the relaxation response, the practice of adaptive coping and cognitions, behaviors necessary to create a healthy lifestyle, and methods of building social support. Throughout the course of intervention, participants were asked to elicit the relaxation response at home each day, through development of a consistent mind-body practice. Practices for achieving healthy lifestyles like mindful eating and physical activity (yoga), daily appreciation journals of people and events they are thankful for, exploration and challenge of patterns of distorted or irrational automatic thoughts were also part of the curriculum. Group exercises promoting humor, empathy, and social support were also used. Sessions followed a regular structure and included time for didactic, experiential, and discussion portions, as well as meditation.	Coping self-efficacy<br>Morale	N/A<br>Psychological well-being
Hero Lab	Sundar et al. (2016), India	At-risk Hindu youth (low-income community)	Improve mental health	Five days a week, 6-month long, interactive program rooted in positive psychology interventions focused on themes that promote well-being (e.g., grit, empathy, hope). The lessons lead to a student-led project, in which participants exercise their strengths and skills in a design-thinking process to ideate, prototype, and launch a project in their neighborhood to better their community. Activities include performance and skits, finding local heroes, gratitude letters, storytelling, community family interviews, games and others.	**Community service project implementation**<br>Happiness<br>Search for meaning of life<br>Presence of meaning in life<br>Grit<br>Empathy<br>Gratitude<br>Optimism/Pessimism<br>Curiosity	**Community well-being**<br>Overall well-being<br>*Character strength*<br>*Character strength*<br>*Character strength*<br>*Character strength*<br>*Character strength*<br>*Character strength*<br>*Character strength*

N/A	Zhou et al. (2016), Hong Kong, China	Community residents of a low socioeconomic status neighborhood	Increase targeted behaviors that enhance family relationships	The community-based participatory research was composed of two interventions aimed at increasing targeted behaviors grouped into three themes (hope, gratitude or open-mindedness), such as setting goals, problem solving, recalling past successes for hope enhancement, and discussion of controversial issues and evaluation of debates on both sides for open-mindedness).<br><br>The basic intervention (BI) and the basic intervention plus planning (BI+P) were given by social workers from participating NGOs to participants and at least one member of their family aged 6 and over. The BI+P had supplemental material to emphasizes enhancement of intention through increasing awareness of outcomes, and planning to promote intention's translation into behavior change. Both interventions were designed to educate as to the positive effects of the targeted behaviors of the chosen theme and to motivate participants to adopt the targeted behaviors in the family context.<br><br>Both structures contained a core intervention session (at least 2 hour long), a booster session (1 hour long, six weeks following the core session), and a social gathering without intervention (three months after the core session) for assessment. Social workers were required to: (a) cover each of the four theme-related behaviors entirely; (b) begin with a brief introduction to the program, followed by an ice breaking activity to engage the participants; (c) engage in interactive activities (competitions, games, group discussions, personal sharing, workshops, and drama) in order to engage members and communicate the core messages; (d) do a brief summary talk to emphasize the importance and/or benefits of these targeted behaviors within their families; (e) cover planning in the BI+P program at the end of the session. The planning included a booklet, attributional discussion, and homework was assigned.	**Attitude towards behaviors (gratitude, hope and open-mindedness)**<br><br>**Intention towards behaviors (gratitude, hope and open-mindedness)**<br><br>**Frequency of behaviors (gratitude, hope and open-mindedness)**<br><br>Self-perceived health<br><br>Family health<br><br>Family happiness<br><br>Family harmony<br><br>"Increased awareness concerning behaviors targeted behaviors"<br><br>"Increased target behaviors"<br><br>"Promoted family relationships"<br><br>"Promoting family happiness"<br><br>"More opportunities for family communication"	***Character strength***<br><br>***Character strength***<br><br>***Character strength***<br><br>Physical well-being<br><br>Physical well-being<br><br>Overall well-being<br><br>Interpersonal well-being<br><br>N/A<br><br>*Character strength*<br><br>Interpersonal well-being<br><br>Overall well-being<br><br>Interpersonal well-being

Supplementary Table 2.

Effectiveness of the programs

Program name	Authors	Effectiveness	Notes
Everyday Matters	Alschuler et al. (2018)	+/-	Results of the evaluation were mixed, with positive results in the primary measure of psychological resilience but most other measures remaining unchanged.
N/A	Amonoo et al. (2021)	-	Evaluation showed that the vast majority of variables measured remained unaffected by the intervention.
Inspired Life Program	Appiah et al. (2020)	+	Most outcome measures evaluated showed positive results.
N/A	Baker & Ballantyne (2013)	+	Qualitative analyses indicated the presence of positive outcomes following the intervention.
N/A	Bartholomaeus et al. (2019)	+/-	Results of the evaluation were mixed, with positive changes on most measures in one sample (carers) but not in the other (older adults).
Grace intervention	Bufford et al. (2017)	+/-	Results of the evaluation were mixed, with positive changes on primary outcome measures of grace and trait self-forgiveness. Different effects, sometimes contradictory, were noted at different time points in the two samples studied.
Lab. I Emotional-Motivational Empowerment	Cantarella et al. (2017)	+	Most outcome measures evaluated showed positive results, including the primary measure of psychological well-being.
N/A	Carson et al. (2010)	-	Evaluation showed that the vast majority of variables measured remained unaffected by the intervention, including the primary outcome measure of gratitude.
Positive Narration Group Intervention — Older Adults	Cesetti et al. (2017)	-	Evaluation showed that the vast majority of variables measured remained unaffected by the intervention. Only one of the six subscales of the primary target outcome of eudaimonic well-being achieved significant results.
Programa Vem Ser	Durgante & Dalbosco Dell'Aglio (2019)	+/-	Results of the evaluation were mixed, with some measures showing positive changes and other remaining unchanged.
Lighten UP!	Friedman et al. (2017; 2019)	+/-	Results of the evaluation were mixed, with some measures showing positive changes and other remaining unchanged. Positive results were found on one of the two primary measures (eudaimonic well-being) and other secondary

			measures, as well as some subscales of instruments used to measure other target outcomes.
Art of Happiness	Greenawalt et al. (2019), Turner et al. (2017)	-	Evaluation showed that the vast majority of variables measured remained unaffected by the intervention.
Happy Family Kitchen Movement	Ho et al. (2020a; 2020b),	+/-	Results of the evaluation were mixed. Qualitative analyses indicated the presence of positive outcomes following the intervention.
Happy Family Kitchen I	Ho et al. (2016a; 2017)	+	Most outcome measures evaluated showed positive results. Qualitative analyses indicated the presence of positive outcomes following the intervention.
Happy Family Kitchen II	Ho et al. (2016b; 2016c), Chu et al. (2018)	+/-	Results of the evaluation were mixed. Qualitative analyses indicated the presence of positive outcomes following the intervention.
N/A	Ho et al. (2014)	+	All outcome measures evaluated showed positive results.
N/A	Hou et al (2016)	-	Evaluation showed that the vast majority of variables measured remain unaffected by the intervention.
Move More, Feel Well: MAPP (Motivation, Audit, and Positive Psychology)	Millstein et al. (2020)	-	Evaluation showed that the vast majority of variables measured remain unaffected by the intervention.
Strength, Hope, and Resource-fulness Program for people diagnosed with Parkinson’s Disease (SHARP-PWP)	Murdoch et al. (2020)	+/-	Results of the evaluation were mixed, with most quantitative measures remaining unchanged. Qualitative analyses indicated the presence of positive outcomes following the intervention.
N/A	Nikrahan et al. (2016)	+/-	Results of the evaluation were mixed. While most measures remained unchanged after the different interventions, there were positive changes in the primary outcome measure of inflammatory marker hs-CRP post-intervention, in some interventions, but not at follow-up.
MAPEG Program	Ramirez et al. (2014)	+	Most outcome measures evaluated showed positive results.

N/A	Rew et al. (2017)	-	Evaluation showed that the vast majority of variables measured remain unaffected by the intervention.
Healthy Aging Mind Body Intervention	Scult et al. (2015)	+	All outcome measures evaluated showed positive results.
Hero Lab	Sundar et al. (2016)	+	All outcome measures evaluated showed positive results. The community service project was also successfully implemented.
N/A	Zhou et al. (2016)	+	Most outcome measures evaluated showed positive results. Qualitative analyses indicated the presence of positive outcomes following the intervention.

Note. Effectiveness of programs is identified as followed: (+) signifies that the program achieved statistically significant results for most outcomes or that positive outcomes were qualitatively identified; (-) signifies that the program achieved statistically non-significant results for most outcomes or that negative outcomes were qualitatively identified; (+/-) indicate mixed results.
